# No consistent effect of plant species richness on resistance to simulated climate change for above- or below-ground processes in managed grasslands

**DOI:** 10.1186/s12898-017-0133-0

**Published:** 2017-06-17

**Authors:** Carsten F. Dormann, Lars von Riedmatten, Michael Scherer-Lorenzen

**Affiliations:** 1 Biometry & Environmental System Analysis, University of Freiburg, Tennenbacher Str. 4, 79106 Freiburg, Germany; 2 Computational Landscape Ecology, Helmholtz-Centre for Environmental Research, Permoser Str. 15, 04318 Leipzig, Germany; 3 Geobotany, Faculty of Biology, Schänzlestr. 1, 79104 Freiburg, Germany

**Keywords:** Climate change manipulation, C-pool, Ecosystem function, N-pool, Productivity, Species richness, Temperate grassland, Vegetation

## Abstract

**Background:**

Species richness affects processes and functions in many ecosystems. Since management of temperate grasslands is directly affecting species composition and richness, it can indirectly govern how systems respond to fluctuations in environmental conditions. Our aim in this study was to investigate whether species richness in managed grasslands can buffer the effects of drought and warming manipulations and hence increase the resistance to climate change. We established 45 plots in three regions across Germany, each with three different management regimes (pasture, meadow and mown pasture). We manipulated spring warming using open-top chambers and summer drought using rain-out shelters for 4 weeks.

**Results:**

Measurements of species richness, above- and below-ground biomass and soil carbon and nitrogen concentrations showed significant but inconsistent differences among regions, managements and manipulations. We detected a three-way interaction between species richness, management and region, indicating that our study design was sensitive enough to detect even intricate effects.

**Conclusions:**

We could not detect a pervasive effect of species richness on biomass differences between treatments and controls, indicating that a combination of spring warming and summer drought effects on grassland systems are not consistently moderated by species richness. We attribute this to the relatively high number of species even at low richness levels, which already provides the complementarity required for positive biodiversity–ecosystem functioning relationships. A review of the literature also indicates that climate manipulations largely fail to show richness-buffering, while natural experiments do, suggesting that such manipulations are milder than reality or incur treatment artefacts.

**Electronic supplementary material:**

The online version of this article (doi:10.1186/s12898-017-0133-0) contains supplementary material, which is available to authorized users.

## Background

The scientific consensus is unambiguous about the role of biodiversity for ecosystem functioning [1]. It is largely based on experiments along species richness gradients from one to tens of species [2]. There is much less experimental attention being paid to the effect of species richness in managed systems, where the range of diversity is different. In managed temperate grasslands, species richness can be as low as five vascular plant species/m^2^, and as high as 60 species/m^2^ [3, 4]. Thus, the gradient in grassland species richness generally does not cover the very low end of diversity, which is included in the design of many experimental studies (Cedar Creek: 1–16: [5]; Biodepth: 1–16: [6]; Jena: 1–60: [7]). Since at very low species richness diversity matters most [2], it is not clear whether results from experimental grasslands directly translate into long-term managed grasslands, that is, whether such controlled experiments have high external validity.

An important effect of plant diversity is the buffering of environmental fluctuations, such as droughts [5]. Under standard management conditions it remains to be investigated whether plant species richness dampens the effect of fluctuations on ecosystem functioning. Central European plant communities are exposed to high inter-annual variability in weather conditions, and different responses of its members will lead to portfolio effects, buffering the effects of environmental variability at the community level [8–12]. The general existence of these mechanisms is beyond dispute, but its relevance for managed systems with at least a moderate number of species (>2) can be questioned. Such systems include most grasslands (used as pastures or meadows) and non-plantation forests.

Plant-species richness in grasslands is determined through a complex interplay of abiotic conditions (e.g. soil type, climate), land use and its history (fertilisation, grazing, management changes) and regional species pool [13]. If a grassland experiences externally induced disturbances, probably all of these factors have some relevance for the system’s response [14]. Understanding how much resistance (the degree to which a variable changes, following a perturbation: [15] is transferred to the system by being more species rich allows us to gauge the importance of biodiversity *relative* to the effect of land management and abiotic factors in grassland systems.

Here, we exposed temperate grasslands in three regions in Germany to two pulse per-turbations simulating climate change: advanced spring and summer drought, representing two opposing effects of climate change on vegetation. Earlier spring will extend the vegetation growth period, potentially increasing total productivity, with knock-on effects on plant phenology and belowground processes. These positives effects may be offset by prolonged drought phases in summer, reducing grassland productivity in the late-summer growth period. Climate-change predictions of precipitation are notoriously uncertain, so our manipulations set out to explore a warm-spring–dry-summer scenario, rather than represent a specific future climate prediction.

Within three land-use types, a realistic but still substantial gradient in plant species richness was present. We measured several above- and below-ground biomass, C- and N-pools to assess ecosystem functioning in two consecutive periods, attempting to test our central hypothesis that plant diversity buffers effects of climatic variability in typical temperate European grasslands. Moreover, we expect different land-use types to respond differently to our climate-change manipulations, thereby revealing how management affects ecosystem resistance. Finally, by assessing ecosystem processes in the vegetation and the soil, we can compare whether biodiversity effects are greater above- or below-ground, linking the presumed buffering mechanism to wider biogeochemical processes.

## Methods

In this study, we imposed a combination of summer drought and an increase in next year’s spring temperature in managed grasslands and assessed the effects on vegetation, soil properties and litter decomposition relative to unmanipulated controls. We selected three grassland management types (pasture, meadow, mown pasture), with five replicates each. Across these 15 sites, each land-use type covers a gradient in plant species richness, which we exploited for testing our hypothesis that plant species richness buffers climate change manipulations. The same setup was replicated across three regions within Germany, the locations of the Biodiversity Exploratories, a long-term research platform into the interrelationships of biodiversity, ecosystem functioning and land-use intensity in grasslands and forests. The three locations differ in climate, geology, management of grassland, plant species richness and soil properties (see below). If results are consistent across the three regions, we can claim to have elicited a generalisable response, despite different pathways of how land use affects plant species richness [16, 17].

### Study regions

The experiment took place in managed grasslands of the three Biodiversity Exploratories [18]. Schorfheide-Chorin (SC) in northeast Germany spans an area of 1300 km^2^. In this young glacial landscape, plots were established on former fens at an elevation of 3–140 m a.s.l. The main soil type is Histosol, rich in organic matter. The annual mean temperature (1981–2010) averages 8–8.5 °C (summer: 18.2 °C; winter: 1.1 °C) and the annual mean precipitation 500–600 mm (driest month: April, 24 mm; wettest month: July, 69 mm). Hainich-Dün (HD) lies in central Germany with an area of 7600 km^2^ and an elevation of 285–550 m a.s.l. on a calcareous bedrock (Vertisol as main soil type), with an annual mean temperature of 6.5–8 °C (summer: 17.7 °C; winter: 1.5 °C) and an annual mean precipitation of 500–800 mm (driest month: April, 24 mm; wettest month: July, 67 mm). The third exploratory Swabian Alp (SA) in southwest Germany extends over an area of 422 km^2^ at an elevation of 460–800 m a.s.l. Annual mean temperature is 6–7 °C (summer: 15.8 °C; winter: −0.4 °C) and the annual mean precipitation 700–1000 mm (driest month: February, 47 mm; wettest month: July, 119 mm). The soil type is Cambisol on a calcareous bedrock with karst phenomena. A detailed description of all three exploratories is given by Fischer et al. [18].

We used five replicate plots of similar soil in each of three grassland management regimes: meadow (m, fertilized), mown pasture (mp, fertilized and unfertilized) and pastures (p, unfertilized; grazed by sheep, horse or cattle). Meadows were traditionally restricted to edaphically extreme sites, where livestock trampling could destroy the vegetation. In recent years, overall reduction in cattle grazing has led to emergence of meadows on all soils, particularly in parcels of land that are small and further away from the farm. Depending on soil type, meadows are mown once or twice per year, but up to four times under heavy fertilisation. Pure pastures are nowadays common only as nature management regime, predominantly grazed by sheep. The typical mown pasture receives a late-season cut to prevent spreading of unpalatable herbs, after livestock (mainly cattle) is moved on to other pastures. There is much variation among the three sites in the timing and intensity of grazing both on mown and pure pastures, but within a site management is relatively consistent. By using replicates of land-use types, we aimed to introduce variability in species richness within each management, thereby reducing the correlation between land use and plant species richness inherent in the setup of the Biodiversity Exploratories.

### Experimental setup of climate change manipulations

Experimental manipulations took place in 2008/2009 and 2009/2010, from the middle of June until the end of July in 2008 and 2009 for the simulated drought treatment and from end of February till end of March in 2009 and 2010 for simulation of increased spring temperatures. Our manipulations thus implicitly include carry-over effects of drought on the spring treatment the following year. Treatments are representing projected qualitative changes in summer drought duration and concurrent changes of the length of the vegetation period. Their length is based on interannual variability of the onset of spring (approximately 4 weeks after begin of snow melt in late February) and length of drought in summer (maximally 6 weeks). We left the rain shelter in place until there were cumulatively 3 weeks of rainfall withheld from the treatment (monitoring the closest German weather service stations for guidance). Thus, all treatments are within the variability witnessed in the region, but aim at representing earlier season and prolonged drought. Constructions were removed when no treatment was applied, but the plot was fenced off for the entire vegetation period, which we do not expect to have substantial short-term impacts. The entire plot was mown in summer after biomass harvest (see below).

**Fig. 1:**
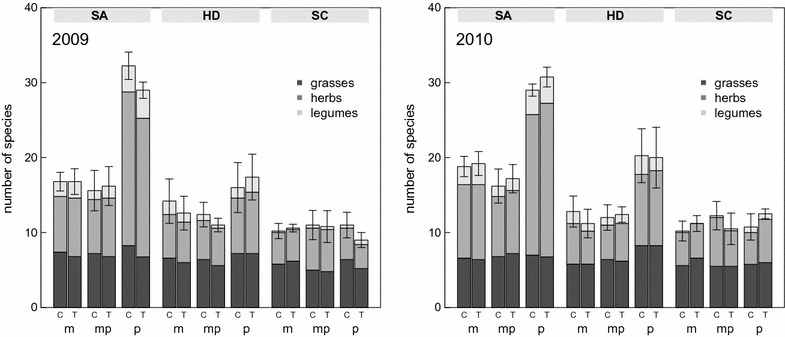
Number of vascular plant species, sub-divided by functional groups, found on control (C) and treatment (T) subplots by land-use type (*m* meadow, *p* pasture, *mp* mown pasture) and year (2009 *left*, 2010 *right*). *Bars* represent mean values ± SEM of the total number of species. Exploratories are referred to as SA (Swabian Alp, south-west Germany), HD (Hainich-Dün, central Germany) and SC (Schorfheide-Chorin, north-east Germany)

Each plot of 5 × 3 m was divided in two subplots, one for the manipulation, the other as control (see Additional file 1: Figure S6). Open-top chambers (OTCs) are commonly used to raise the temperature in climate change experiments with minor effects on gas exchange and ambient precipitation [19]. To increase temperature in early spring, we placed OTCs (2 × 3 m and 1.4 m height) on each treatment subplot. The OTCs were constructed from four PVC tube arches, which supported the 0.2 mm thick greenhouse plastic sheet (UV 5 Coex-foil made of ethylene vinyl acetate copolymers, Folitec Agrarfolien-Vertriebs GmbH, Westerburg, Germany) up to a height of 1.2 m (see [20] Fig. 1D for a similar design; Additional file 1: Figure S7). Soil temperature (10 cm depth) and air temperature (10 cm height) were recorded every half an hour by aluminium-foil-shielded temperature sensors (Thermochron iButton, Maxim Integrated Products Inc., Sunnyvale, CA, USA).

For the drought manipulation the same treatment plots were covered by rain shelters constructed from the same construction as the OTCs, but using the greenhouse foil as a top cover. Air was thus allowed to freely circulate underneath the roof. The air temperature was again recorded by temperature sensors every 30 min at a height of 10 cm. Soil moisture during the drought experiment was measured every 30 min in a depth of 10 cm by a moisture sensor (ECH2O, type EC-5, Decagon Devices Inc., Pullman, USA) and recorded by a data logger (Em5b, Decagon Devices, Pullman, WA, USA). In spring, no soil moisture measurements were possible because sensors were removed from the soil over winter and could not be re-inserted into the frozen ground until later in the season.

Open-top chambers increased spring temperature by 0.5 °C in the air and 0.35 °C in the soil (see Table 1), in a period of absolute temperatures between −5 °C at night and 15–20 °C during the day (Additional file 1: Figure S1). In summer, rain shelters had a warming effect (Table 1) as pronounced as in spring, but at much higher overall temperatures (12–35 °C). Soil moisture during this period was reduced under the shelter by 8–20%vol, with substantial differences between exploratories (Table 1; Additional file 1: Figure S1). In all sites, soil moisture in the control plots averaged to 30–40%vol, but soil moisture was most affected by drought treatment in the Swabian Alp. Both at the SA and HD sites, the drought treatment presumably resulted in soil water potential being more negative than the permanent wilting point (PWP, at pF = 4.2) as estimated from soil texture data with high clay and silt contents. At the SC site with sandy soils, however, soil water potential was still above the PWP (see Additional file 1: Figure S1).

**Table 1:** Differences (manipulation—control, given as mean ± standard deviation) in temperature and soil moisture during the different phases of the experiment

	Air temperature difference (°C)			Soil temperature difference (°C)			Soil moisture difference (%vol)		
	SA	HD	SC	SA	HD	SC	SA	HD	SC
Period 1									
2008 summer drought	1.15 ± 2.179	0.32 ± 2.178	0.53 ± 2.433	0.84 ± 1.617	−0.032 ± 0.494	0.075 ± 0.623	−20.1 ± 6.53	−9.3 ± 3.67	−7.8 ± 2.40
2009 spring warming	0.69 ± 1.310	0.39 ± 0.607	0.36 ± 0.619	0.45 ± 0.818	0.30 ± 0.585	0.20 ± 0.464	*	*	*
Period 2									
2009 summer drought	0.62 ± 1.333	1.30 ± 2.694	0.64 ± 1.332	−0.30 ± 0.601	−0.50 ± 1.420	−0.19 ± 0.452	−20.0 ± 5.24	−18.8 ± 12.48	−7.5 ± 2.91
2010 spring warming	1.00 ± 1.474	0.47 ± 0.713	0.60 ± 0.866	0.68 ± 0.777	0.28 ± 0.235	0.39 ± 0.204	*	*	*

Exploratories are referred to as *SA* Swabian Alb, *HD* Hainich-Dün, *SC* Schorfheide-Chorin

* Indicates that no sensor were employed because the soil was frozen

### Vegetation recording

In April 2009 and 2010 vegetation recordings were carried out using a 50 × 50 cm frequency frame with a 5 cm grid on each sub-plot. Summer recordings in a pilot study lacked several of the early spring flowers and detected fewer species. These subplots were established centrally in the plot, 1 m from the northern end of the plot’s border to reduce edge effects. Subplots were marked by metal pegs in the ground to record in the exact same site in the following year. Plant species were tallied for each grid cell, yielding percentage cover values [21]. Vegetation recordings in 2009 were used to compute Shannon diversity for each plot.

### Plant biomass harvest and analysis

Above-ground biomass was harvested in an area of 50 × 50 cm next to each vegetation-recording subplot before the summer drought treatment 2009, after the summer drought treatment 2008 and 2009 and after the spring warming treatment 2010, by clipping all shoot material above the soil surface and sorting into functional groups (grasses, herbs and legumes). The treatment extended the sampled area on all sides, obviating the necessity of trenching the plot. Treatments showed marked effects with no sign of leakage to outside the treated area.

To determine root growth during the summer drought treatment, we established three in-growth cores [22] per subplot. These 5-cm diameter 10-cm long frames were inserted into the soil using a soil corer and filled with sieved local soil. Plant roots growth into the in-growth core volume was sampled using the soil corer, severing roots around the in-growth core. Root material was obtained by washing in-growth cores over sieves (0.5 cm and 0.2 mm mesh size). This procedure was repeated in summer 2008 and 2009, yielding root production values for the summer drought experiment.

Above- and belowground plant material was dried at 70 °C for 48 h, then weighed to the nearest mg. For C and N analysis, dried plant material was ground in a ball mill and a subsample of 5 mg was processed in an elemental analyser (PerkinElmer 2400 Series II, PerkinElmer, Waltham, MA, USA).

### Soil sampling and analysis

20 g bulk soil was collected by taking five samples of the upper 10 cm randomly from each subplot. 5 g soil were dried at 105 °C for 48 h to determine gravimetric water content. A subsample of 5 mg was analysed for %C and %N after grinding in a ball mill. To measure soil nitrogen content, 5 g soil were extracted with 50 ml of 1 M KCl solution on a shaker for 20 min. After filtering (Black Ribbon filter, Grade 589/1: 12–25 mm, Whatman Ltd., Maidstone, UK) extract was analysed for C and N with vario MAX CNS (Elementar, Hanau, Germany). The percentage of soil organic matter was determined by loss on ignition of 1 g oven-dried soil at 500 °C for 24 h.

### Data analysis

Our main interest was to test the interaction of species richness, our manipulation and land use on the various response variables across the three sites as measured in summer. Differences between the two consecutive phases of the experiment test for consistency of treatment effects, and indirectly allow us to compare effects to inter-annual variability. We regarded cumulative treatment effects as negligible after only two seasons, given the visual similarity of treatment and control *before* the rain shelters were put up. No lasting effect of spring warming was discernible. Spring data analysis of above-ground biomass reflects response to earlier warming, and in our opinion merely demonstrates the effectiveness of this treatment.

Biomass, vegetation and soil C and N pools were expressed on a per m^2^ basis by extrapolation from the sampled area. We used Linear Mixed Effect Models (lme: [23] with plot identity as a random factor to test the effects of climate change manipulation, land-use management and identity of the three exploratories (as fixed effect) on species richness, plant diversity, functional diversity and on change of plant productivity and C and N pool.

To test our hypothesis that species-rich grasslands will be better able to resist the effects of our warming (spring) and drought (summer) manipulation, we analysed the difference in biomass between treatment and control as log-response ratio (LRR = log(treatment) − log(control)), with approximately normal distribution of residuals. Values above 0 indicate a stronger response of the treatment than the control. In addition to exploratory and land-use type we also included species richness (and interactions) as predictor in the model.

Across and within the exploratories, plant species richness varies with land-use type, but with substantial scatter (e.g. [24]. We thus first analyse the correlation between exploratories, land-use type and plant community composition and richness in our study plots. Results largely refer to spring 2010 and summer 2009, i.e. after two periods of the respective treatment, to represent the largest effects in our study.

Effects on vegetation composition were analysed using Canonical Correspondence Analysis with package *vegan* [25]. All statistical analyses were conducted using R version 3.0.3 [26]. R-code for analysis and figures is available on request from the corresponding author. The data are available freely from the Biodiversity Exploratories data base at https://www.bexis.uni-jena.de (data set number 20186).

## Results

### Species richness, diversity and composition

The number of recorded vascular plant species and Shannon diversity showed a significant interaction between the three study regions and land-use types (species richness *S*: Fig. 1; Table 2; Shannon diversity *H*: Additional file 1: Figure S3). The significant differences of species richness between the land-use types are due to the varying quantity and richness of herbs and, to a lesser degree, legumes, while grass richness was very similar (Table 2). The pastures in the Swabian Alp, in contrast to the pastures of the two other exploratories, are located in semi-dry grassland and are grazed by sheep, which explains the noticeable higher number of species (32 species compared to only 17 and 11 in Hainich-Dün and Schorfheide-Chorin, respectively; Fig. 1). Legumes were particularly scarce in the Schorfheide-Chorin, both in terms of species numbers and their biomass (see below). Neither total nor functional-group species richness differed significantly between treatments (all P > 0.085; Table 2), but by the end of the experiment (2010), Shannon diversity showed a consistent and significantly positive treatment effect (F_1, 121_ = 5.50, P < 0.05; Additional file 1: Figure S3). Finally, plant species composition differed substantially among exploratories and land uses, but was affected only very mildly by our treatments (Additional file 1: Figure S2).

**Table 2:** Mixed-effect model analysis of *species richness* (total as well as of the three functional groups) to exploratories, land use, treatment in the two experimental periods with plot-ID as random effect (see Fig. 1)

	numDF	Total		Grasses		Herbs		Legumes	
		denDF	*F*	denDF	*F*	denDF	*F*	denDF	*F*
Period 1									
Explo	2	35	19.8***	39	4.79*	35	21.3***	39	10.6***
LUT	2	35	7.81**	39	1.40^n.s.^	35	10.2***	39	3.46*
Treatment	1	43	2.00^n.s.^	43	7.08*	43	0.263^n.s.^	43	0.328^n.s.^
Explo:LUT	4	35	4.78**			35	9.33***		
Period 2									
Explo	2	32	23.0***	36	2.04^n.s.^	32	29.5***	36	13.9***
LUT	2	32	13.2**	36	1.60^n.s.^	32	17.5***	36	4.19*
Treatment	1	40	0.738^n.s.^	40	2.26^n.s.^	40	0.603^n.s.^	40	0.122^n.s.^
Explo:LUT	4	32	3.16**			32	6.29***		

Numerator degrees of freedom (numDF) are constant across all models, while denominator degrees of freedom (denDF) change with the number of parameters in the model. Significance of *F* value of *P* < 0.001, <0.01, <0.05 and not significant are indicated by ***, **, * and ^n.s^, respectively. Models were simplified manually until only significant effects remained (or effects marginal to interactions). Design variables were always included in the model, irrespective of significance. LUT and Explo refer to land-use type and exploratory region, respectively

### Productivity

**Fig. 2:**
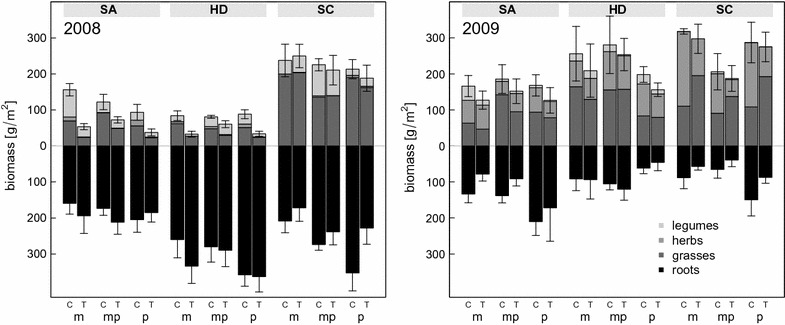
Standing above- and below-ground biomass in summer, subdivided by functional groups. *Bars* represent mean values ± SEM of the total above-ground biomass, below-ground biomass respectively. See Fig. 1 for abbreviations

Treatment manipulations showed significant effects on above- and below-ground plant productivity measured in summer across all exploratories and land-use types (F_1, 81_ = 46.3, P < 0.001; Fig. 2; Table 3). Above-ground biomass was sometimes substantially reduced (e.g. in the Swabian Alp), while root increment showed initially an inconsistent pattern (as indicated by the marginally significant treatment × exploratory-interaction in Table 3; Fig. 2 left; F_2, 82_ = 2.82, P < 0.1). At the end of the second season (2009), also below-ground biomass was uniformly reduced due to the summer drought treatment (F_1, 78_ = 4.88, P < 0.01; Fig. 2 right; Table 3). In contrast, above-ground biomass remained at control levels in 2009, and none of the three functional groups displayed a significant response to the treatment (Table 3).

**Table 3:** Analysis of *summer biomass* (i.e. harvested around peak biomass) in periods 1 and 2 (see Fig. 2). See Table 2 for explanation of symbols

	df	Total		Grasses		Herbs^a^		Legumes^b^		Roots	
		SS	*F*	SS	*F*	SS	*F*	SS	*F*	*SS*	*F*
Period 1											
Explo	2	28.7	90.6***	39.8	67.6***	18.0	2.69^†^	91.7	9.44***	4.14	15.7***
LUT	2	1.46	4.60*	0.544	0.924^n.s.^	33.3	4.97**	79.1	8.13***	1.06	4.02*
Treatment	1	7.33	46.3***	7.38	25.0***	20.2	6.02*	23.6	4.85*	0.0260	0.198^n.s.^
Treatment:Explo	2	2.69	8.51***	2.31	3.92*					0.742	2.82^†^
LUT:Explo	4			4.20	3.57*			85.2	4.38**		
Residuals		12.8	(df = 81)	22.7	(df = 77)	278	(df = 83)	384	(df = 79)	10.78	(df = 82)
Period 2											
Explo	2	5.16	10.4***		3.98*	5.24	5.58**	27.85	4.29*	4.93	4.91**
LUT	2	0.179	0.360^n.s.^	0.797	0.846^n.s.^	2.00	2.14^n.s.^	6.25	0.962^n.s.^	0.366	0.365^n.s.^
Treatment	1	0.575	2.31^n.s.^	0.020	0.0422^n.s.^	1.06	2.25^n.s.^	8.58	2.65^n.s.^	4.88	9.73**
Treatment:Explo	2			2.90	3.08^†^						
LUT:Explo	4					5.51	2.93*			8.19	4.08**
Residuals		20.4	(df = 82)	37.7	(df = 80)	36.6	(df = 78)	178	(df = 55)	39.12	(df = 78)

Biomass data were log-transformed to achieve homogeneity of variances. Models were manually simplified until all model terms were (marginally) significant. Design effects were always kept in the model. LUT and Explo refer to land-use type and exploratory region, respectively

^†^Indicates a *P* value between 0.05 and 0.1

^a^A constant value of 0.1 was added to all response values in 2008

^b^Response values of 2008 were square-root, rather than log-transformed; for 2009, a constant value of 0.1 was added

Early spring warming led to increased biomass across all exploratories and land-use types (Additional file 1: Figure S4), despite having had reduced biomass at the end of the previous summer. Grasses profited most from elevated temperature manipulations.

**Fig. 3:**
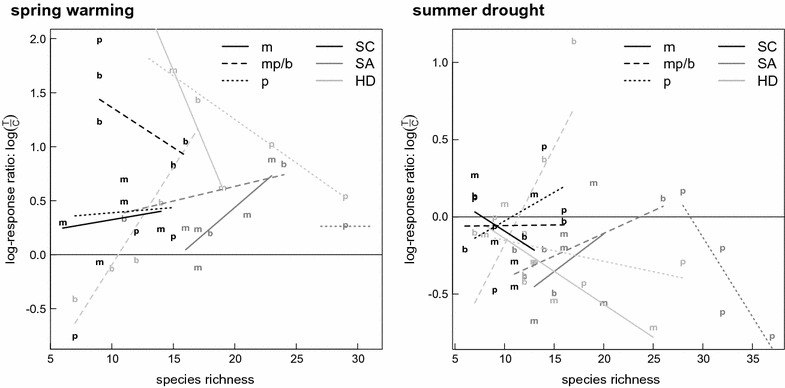
Contrast in biomass between treatment and control at the end of a temperature (spring 2010 *left*) and rainfall (summer 2009 *right*) manipulation period, expressed as log-response ratio. *Negative values* indicate a negative treatment effect, 0 indicates no difference between treatment and control. *Lines types* and *letters* refer to land-use types (where “b” stands for “both”, i.e. “mown pastures”), *grey shades* represent the three different exploratories. In spring, species richness had a small but significant effect, while neither land-use type, exploratory nor their interaction(s) were significant. In summer, the three-way interaction of species richness, land-use type and exploratory was significant (see Table 3)

Although our design was sensitive enough to detect a significant three-way interaction in summer, and effects of species richness in spring (Table 4), LRR showed no interpretable pattern with respect to species richness. The positive effect of species richness for the response to earlier spring was very small and contributed less than 4% to the variance in the data (F_1, 26_ = 4.77, P < 0.05). Responses to combined spring and summer manipulations display huge scatter in LRR. Depending on land-use type and exploratory, positive, negative and no correlations with species richness were observed, accompanied by a significant four-way interaction (F_4, 25_ = 2.90, P < 0.05; Fig. 3; Table 4).

**Table 4:** Analysis of *log*-*response ratios* $\left( {{ \log }\frac{\text{treatment biomass }}{\text{control biomass}}} \right)$ for treatment effects in spring 2010 and summer 2009 (see Fig. 3). See Table 2 for explanation of symbols

	df	Spring^a^		Summer	
		SS	*F*	SS	*F*
Explo	2	0.265	0.347^n.s.^	0.392	2.02^n.s^
LUT	2	0.250	0.327^n.s.^	0.260	1.34^n.s^
Species richness^b^	1	1.77	4.77*	0.0177	0.182^n.s^
Explo:LUT	4			0.342	0.883^n.s^
Explo:species richness	2			0.0544	0.281^n.s^
LUT:species richness	2			0.764	3.94*
Explo:LUT:species richness	4			1.125	2.90*
Residuals		9.03	(df = 26)	2.42	(df = 25)

Models were manually simplified until all model terms were significant. Design effects were always kept in the model. SS were computed sequentially (type I). LUT and Explo refer to land-use type and exploratory region, respectively

^a^Species richness was modelled as log(species richness) in the spring analysis. Using a linear scale yielded similar, but non-significant effects of species richness

^b^Estimate for the spring effect of log(species richness): 0.792 ± 0.363; partial model *R*_adj_^2^ = 0.036

### Vegetation C and N pools

**Fig. 4:**
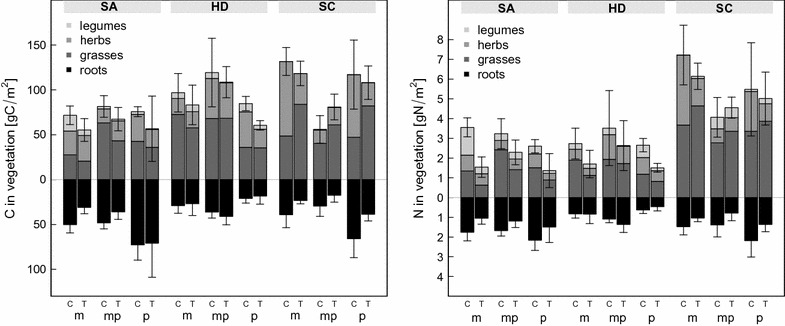
Carbon and nitrogen pools of vegetation biomass in late summer 2009 in dependence of exploratory, land-use type, treatment and functional groups. *Bars* represent mean values and ±SEM (N = 5)

Land use had no consistent effect on carbon pools, and our experimental manipulations manifested themselves with similar magnitude in species-rich pastures and less species-rich meadows. After 2 years of our manipulation treatments, carbon pools in the vegetation were overall reduced by about 10%, both above- and below-ground (Fig. 4 left; F_1, 74_ = 5.43, P < 0.05 and F_1, 75_ = 0.438, P > 0.1, respectively). Differences between exploratories were substantial, with the Swabian Alp having the lowest carbon pools and Schorfheide-Chorin the highest, although this overall pattern did not hold for mown pastures (significant land use–exploratory interaction: F_4, 74_ = 2.84, P < 0.01; see Additional file 1: Table S1).

The pattern for nitrogen in vegetation was similar to that of vegetation carbon (Fig. 4 right). The above-ground treatment manipulation effect was even more pronounced here (F_1, 74_ = 7.43, P < 0.01), as was the difference between Schorfheide-Chorin and the other two exploratories. The significant land use-exploratory interaction (F_4, 74_ = 4.50, P < 0.01) was here due to mown pastures having higher N-pools in SA and HD, but lower in SC (Fig. 4 left). In line with responses of functional group biomass, the C- and N-pools of legumes was most affected by our manipulations, followed by that of herbs.

### Soil carbon and nitrogen

**Fig. 5:**
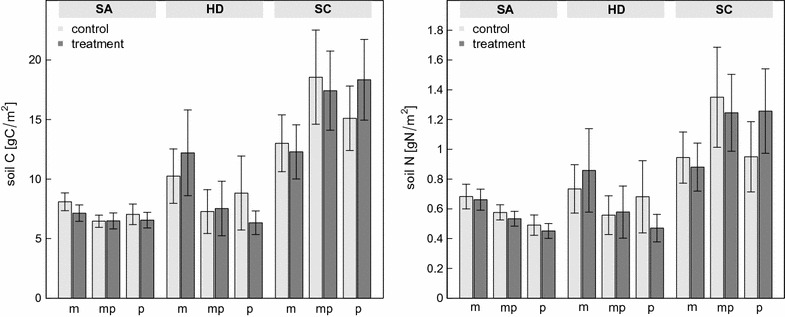
Soil carbon and nitrogen pools (top 10 cm) in late summer 2009 in dependence of exploratory, land-use type, treatment and functional groups. *Bars* represent mean values and ±SEM (N = 5)

Despite the effect of the treatment on C and N in plant biomass, we could not detect changes in the pool of soil C and N due to temperature or rainfall manipulation (Fig. 5, P > 0.48). As for the vegetation C- and N-pools, there were significant differences between the exploratories and land-use types (F_4, 75_ = 3.07, P < 0.05 and F_4, 73_ = 2.83, P < 0.05, respectively), with Schorfheide-Chorin having highest C- and N-levels. The interaction is due to higher C- and N-values for meadows relative to (mown) pastures in SA and HD, but the opposite pattern in Schorfheide-Chorin.

Soil-N correlated significantly with plant species richness, but differently for each exploratory (Exploratory-log(richness) interaction: F_2, 73_ = 5.81, *P* < 0.01; Additional file 1: Figure S5 left). Re-scaling the response within sites suggests no correlation (Additional file 1: Figure S5 right).

## Discussion

Our short-term experimental manipulation of temperature and precipitation led to clearly detectable responses in vegetation biomass, both above- and below-ground, for the three functional groups. However, these responses were idiosyncratic across years, regions and land-use types, and no *consistent* correlation with species richness was detectable. Indeed, only for above-ground biomass and soil N did we detect an effect of plant species richness at all, while vegetation and soil C were responded to treatment, land use and location alone (Additional file 1: Table S1). We hence conclude that species richness does not generally increase the ability of these systems to buffer short-term climate fluctuations in our grassland systems, but that its effect depends greatly on the edaphic and management context. The fact that we observed a significant three-way interaction between exploratory, LUT and species richness on log-response-ratios (Fig. 4) shows that our experimental design was sensitive enough to detect such effects. It was the idiosyncratic response across region-land-use type combinations that led us to reject a consistent buffering effect of plant species richness (see also [27]. More specifically, soil type, climate and land use are processes that in our system affect productivity and biogeochemical processes in grasslands more than species richness per se (in contrast to [28].

The design of the Biodiversity Exploratories uses land-use types to realise a gradient in plant species richness [18]. Indeed, land-use type is a significant predictor for species richness of our plots. For this experiment, however, we replicated the same land-use type five times in an attempt to break this strong association. As a consequence, land-use type did not emerge as particularly strong predictor of species richness (Table 1), opening the way for analysing the additional effect of plant species richness (polyserial correlation between land-use type and species richness is only ρ = 0.325). We believe that through our experimental design and the apparently sufficiently sensitive measurements we would have been able to detect a consistent ecologically relevant buffering effect of plant species richness against imposed drought and warming, if it existed.

Across the three regions, species richness gradients differed greatly (e.g. Fig. 4). Extensive sheep grazing led to high species richness in the Swabian Alp, while the rich, organic soils of Schorfheide-Chorin had very low species richness. This is in line with analyses by Socher et al. [16, 17], who report differential effects of management for the three exploratories: the diversity-promoting effect of grazing in the Swabian Alp is inverted into a negative effect in Schorfheide-Chorin. This may be due to grazing disturbance being at a small spatial scale, increasing dominance of tall species in fertile grasslands [29]. We cannot resolve whether it is the regional conditions that led to different correlations between species richness and buffering ability (Fig. 4) or because this finding is due to the fact that the regions cover different ranges along the species-richness gradient. To address this question, the experiment could be repeated on sites specifically selected to yield similar richness ranges in all three regions.

Beier et al. [30] point out that the approach we have chosen (“multi-factor application”) inevitably confounds the effects of spring warming and summer drought. This is the price to pay for manipulating a specific scenario (or as they call it, the “inevitable dilemma”). The way spring warming and summer drought act in combination is not obvious. Spring warming led to an earlier growth, which could lead to an overall earlier season of unchanged length. We found no visual evidence for shifted phenology in summer, and also Reyes-Fox et al. [31] showed that warming extends, rather than moves, the season in a temperate grassland. Our experiment is insufficient to tease apart the different effects, and how they may accumulate over time.

The interplay of species richness and drought, in particular, has received substantial attention in the ecological literature. To better place our results in this context, we differentiate studies along two axes: (1) whether drought was manipulated or occurred naturally, and (2) whether diversity gradients were experimentally established or naturally realised in the field. In all of the resulting four combinations one can evaluate the importance of biodiversity for buffering drought effects. Our experiment (drought manipulation in natural diversity setting) showed little ability to buffer drought. This is consistent with most similarly designed studies [32–37], which report no biodiversity effect on drought resistance. Furthermore, also drought manipulations in experimental diversity gradients, which are rare, largely found no buffering effect [38–40], but see Kreyling et al. [41] for some buffering, discussed below). This is in contrast to all findings from measurements taken under naturally occurring drought. Plant species richness reduced the effect of drought under natural diversity settings [5, 42, 43] as well as under experimental biodiversity gradients (reviewed in [44, 45]. Natural diversity settings have the distinct disadvantage of confounding richness and environment. If the process that leads to higher plant diversity is also responsible for the system’s resistance to drought, one cannot attribute resistance exclusively to plant diversity (see e.g., [46]). The predominant lack of buffering in drought manipulations even on experimental diversity gradients suggests a different reason, however.

This surprising but rather important finding has apparently not been noticed before, and it demands an explanation that is beyond the scope of this study. An obvious explanation would be that natural droughts are much more severe than manipulations. The EVENT experiment is more radical by simulating a 100-year drought, and indeed finds evidence for buffering of community productivity [39, 41], but not in other responses (reviewed in [47]). More studies need to create such strong manipulations to test whether this is a general pattern. As another potential explanation, we speculate that drought manipulations, typically by rain-out roofs, may have treatment artefacts [48] that limit the ability of the vegetation to compensate for loss in productivity of dominant species. For example, even if roof artefacts did not affect community biomass, they altered light quality [49, 50] and may prevent light-demanding drought-tolerant species to gain from competitive release.

In conclusion, our experiment detected an influence of land use, site conditions and species richness and their interactions on the way vegetation and soil C- and N-pools respond to spring warming and summer droughts. This is largely due to manipulations having opposing effects on plant growth, at least in the short term. We failed to find a consistent effect, however, indicating that species richness per se does not contribute substantially to grassland resistance to climate-change manipulations. We speculate that this can be attributed to the relatively high number of species even at low richness levels in our study system, which already provides the complementarity required for positive biodiversity–ecosystem functioning or biodiversity–stability relationships.

## Additional file

**Additional file 1.** Additional figures and table.
