# Supplementary material for: No consistent effect of plant species richness on resistance to simulated climate change for above- or below-ground processes in managed grasslands
Source: BMC Ecol. 2017 Jun 17;17:23. doi: 10.1186/s12898-017-0133-0 (PMC5473966; doi:10.1186/s12898-017-0133-0)
Supplement: Supplementary file 1 — Additional file 1. Additional figures and table. [file 12898_2017_133_MOESM1_ESM.docx]

1.
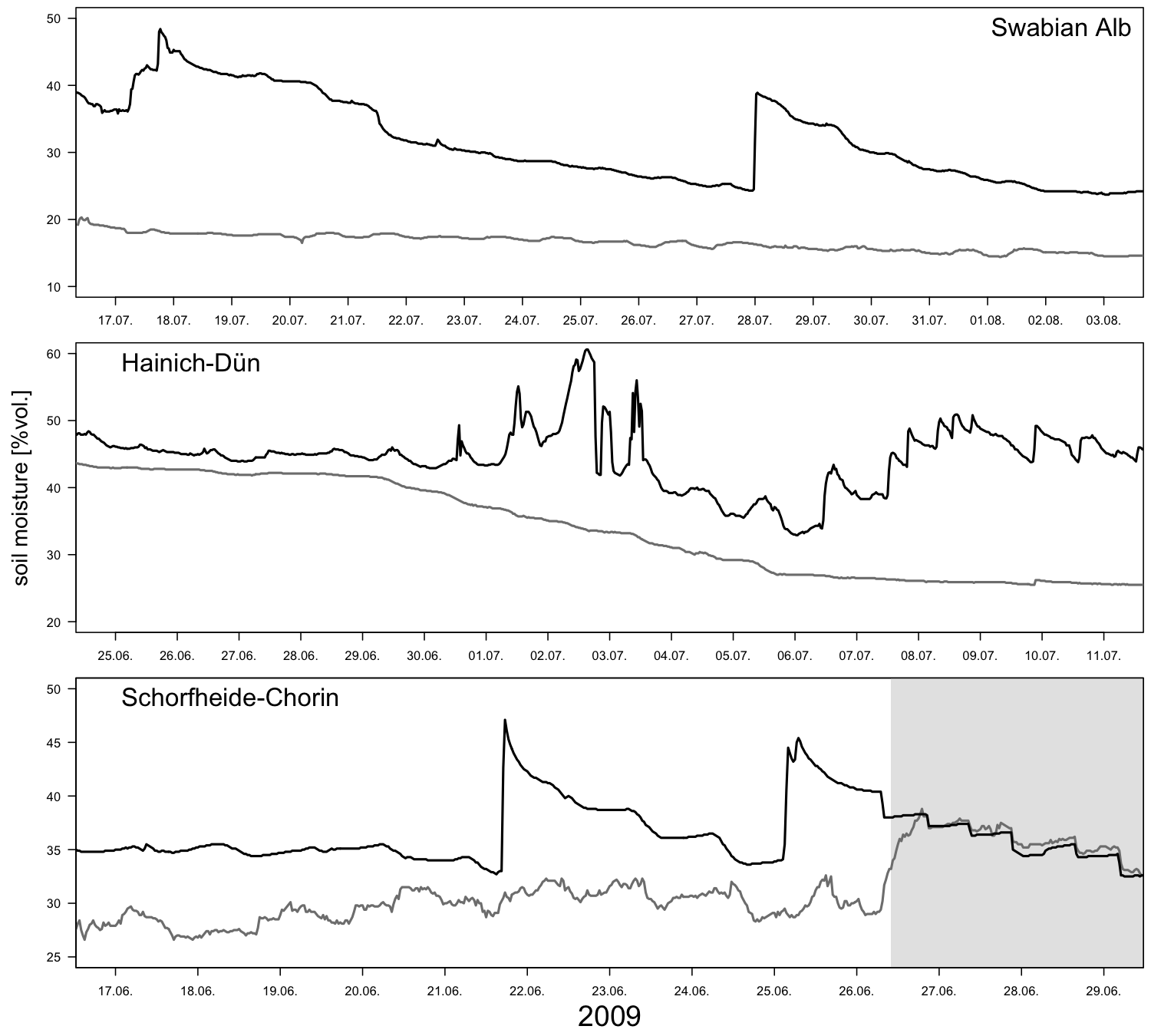

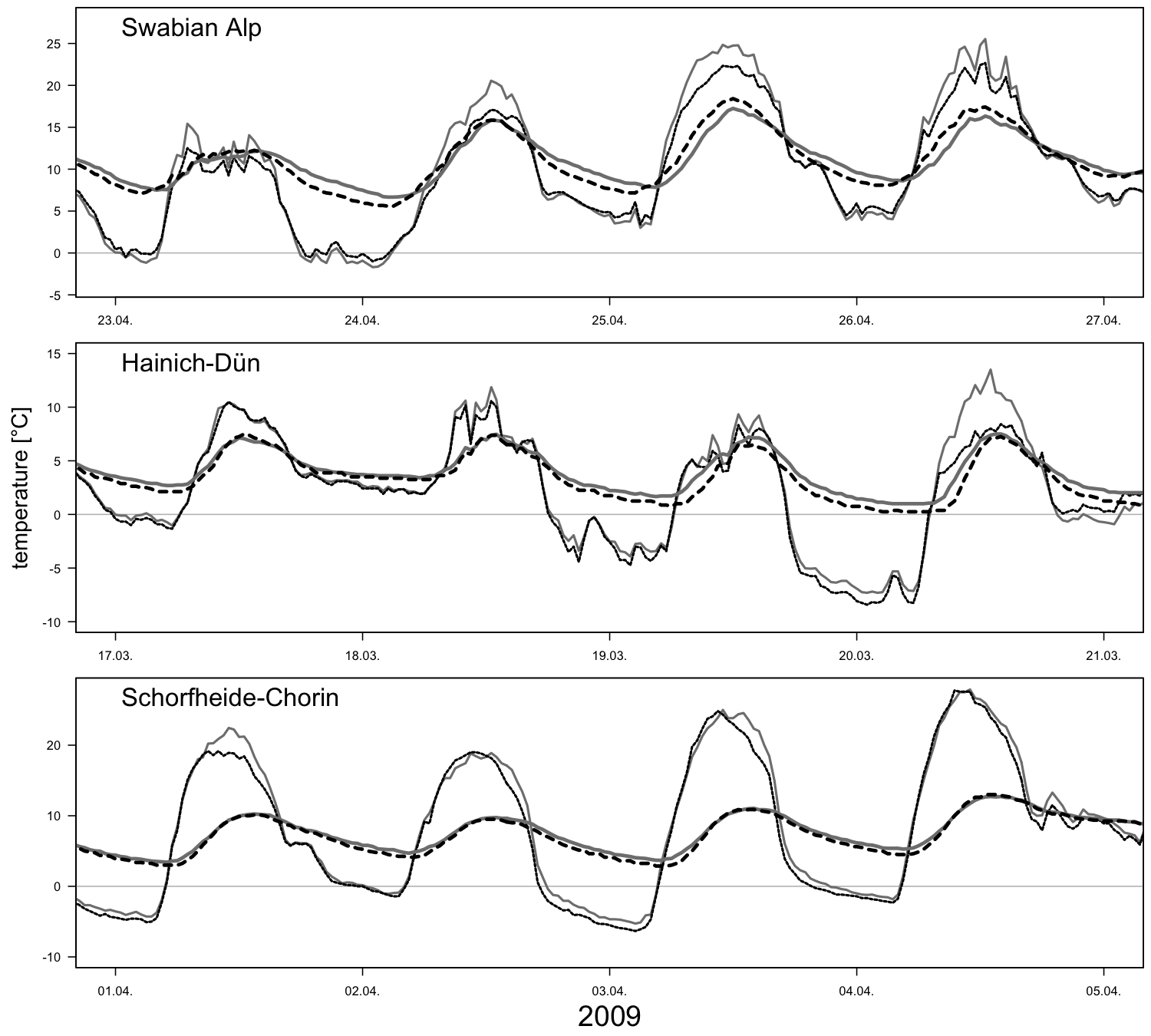
Supplementary material

**Fig. S1**. *Left*: Exemplary temperatures (thin lines: air, thick lines: top soil temperature) during spring 2009 in warmed and ambient plots some weeks after begin of the manipulations. Solid grey lines are warmed plots, dashed black lines ambient. Each line is the average of three plots per exploratory. Soil temperatures show a time delay of several hours and a much buffered response to the diurnal rhythm. Differences between treatment and control were similar in air and soil, however. *Right*: Exemplary soil moisture curves towards the end of summer 2009 drought treatments (grey: rain shelter, black: control). Spikes in the black lines indicate rainfall events. Permanent wilting point (p>4.2) is at around 30%vol for the upper two panels and at 15%vol for Schorfheide-Chorin. Shaded area marks end of manipulation.


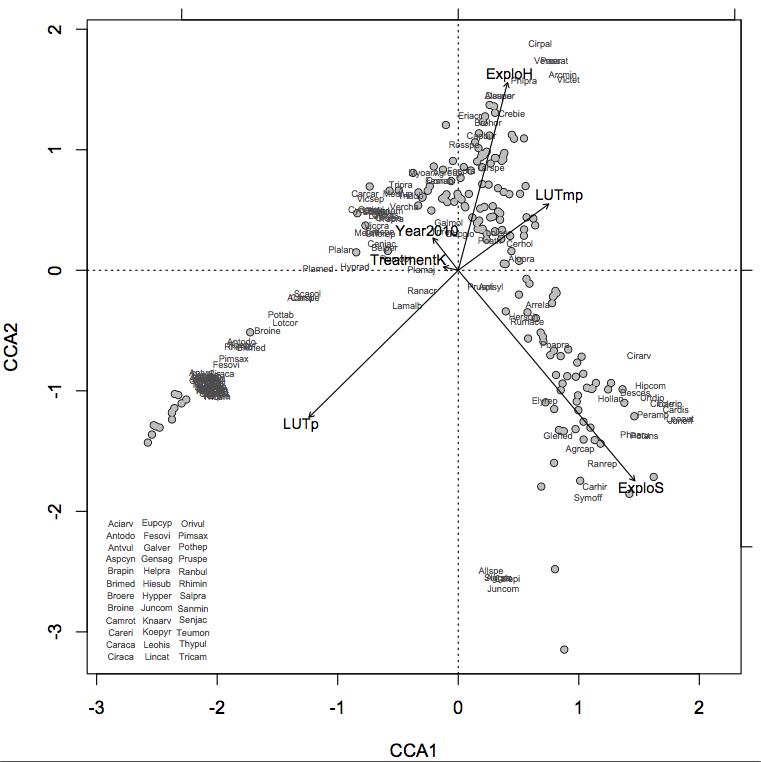


**Fig. S2**. CCA triplot of vegetation composition. Exploratories, land use and treatments are depic­ted as arrows, regions as circles and species by their abbreviated name (first three letters of genus and epithet). The cluster of pasture species at the left is resolved by the name inset, bottom left. The exploratory Schorf­heide-Chorin (“S”) and the pastures are characterised most clearly (and significantly under permutation test: *P* < 0.01) by species composition, while year and treatment had only very small and insignificant effects (*P* = 0.19 and 1.00, respectively). Axes 1 and 2 explain 6.2 and 3.7% of the total inertia (but contribute 43 and 26% to the explanatory power of all axes).

**Fig. S3**. Shannon's diversity (H) by year, exploratory, land use and treatment. The (statistically sig­nificant: *p* < 0.001) difference between years is due to an earlier season in 2009, particularly in Schorfheide-Chorin and Hainich-Dün (significant interaction between exploratory and year, *p* < 0.01).

numDF denDF F-value p-value

(Intercept) 1 121 4073.960 <.0001

Explo 2 40 10.220 0.0003

LUT 2 40 9.303 0.0005

Treatment 1 121 5.500 0.0206

Year 1 121 84.478 <.0001

Explo:Year 2 121 5.080 0.0076

**F
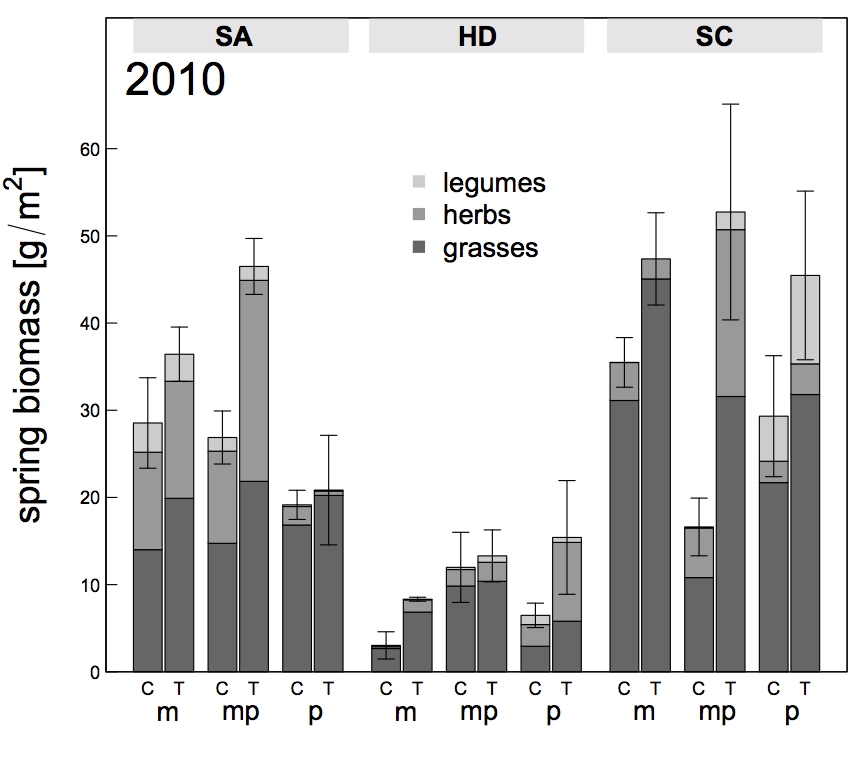
ig. S4**. Biomass in spring 2010 in the different treatments. Differences between treatments and exploratories were significant (F_1, 53_ = 4.65*** and F_2, 53_ = 22.6***, respectively, on log-transformed data), those of land-use type depended on exploratory (F_4, 53_ = 2.59*), but there was no effect of species richness (F_1, 53_ = 0.24 for the main effect, no significant interaction).


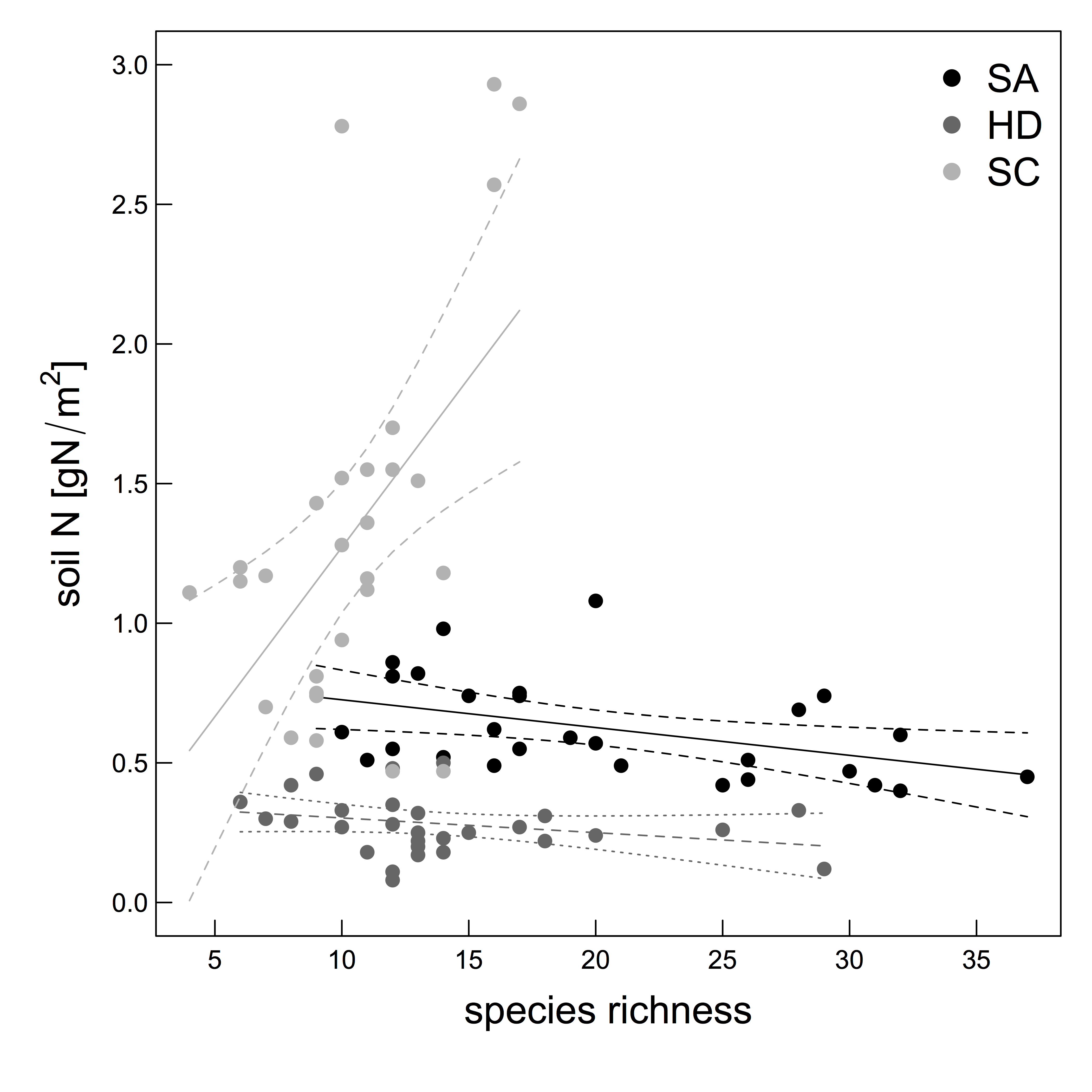

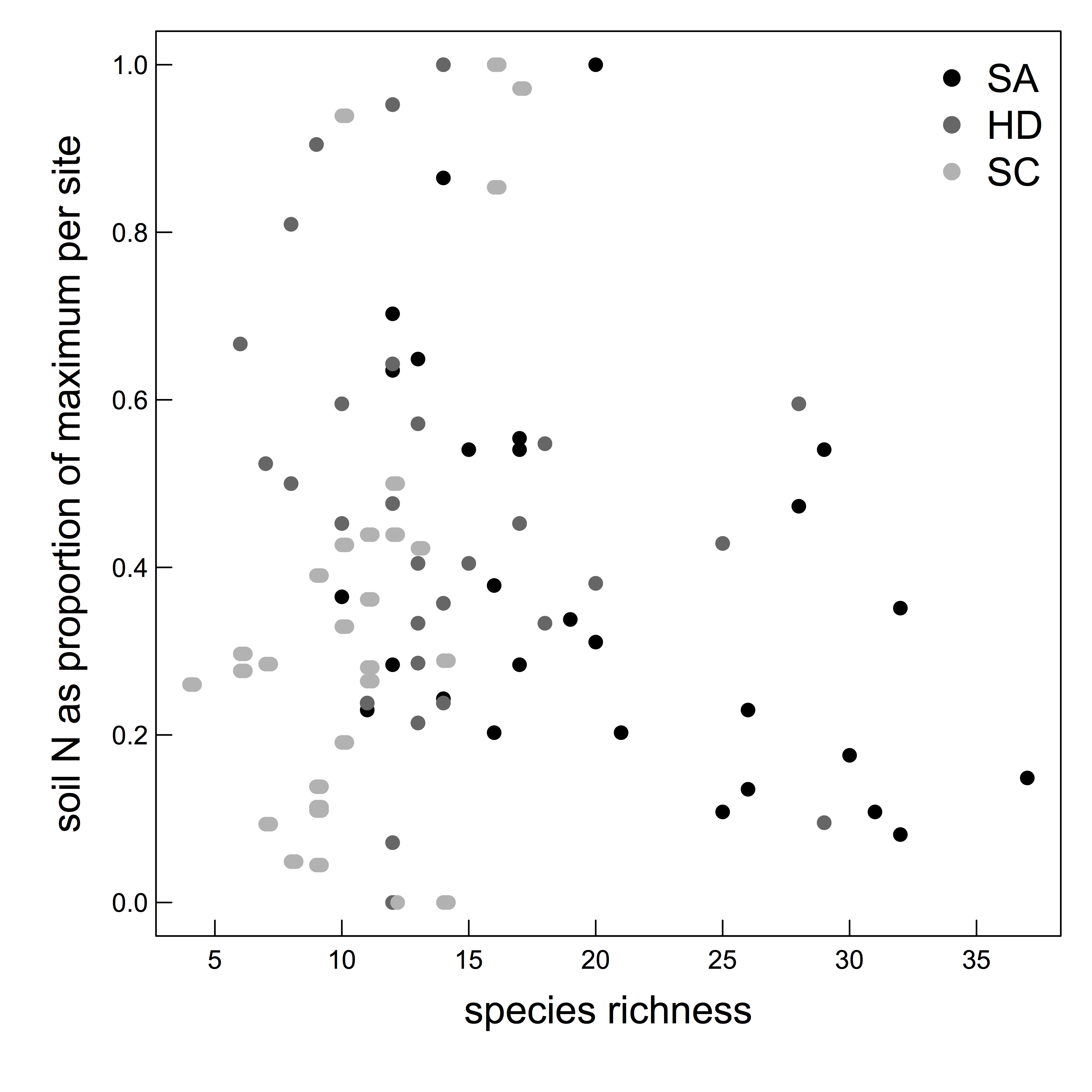


**Fig. S5**. Soil nitrogen content (*left*) as a function of species richness for the three exploratories. This interaction is significant even after correcting for land-use types. Species richness correlates with soil N in Schorf­heide-Chorin (positively) and Swabian Alp (negatively), but not in Hainich-Dün. After ranging the values between min and max for each region (*right*), the pattern sug­gests no relationship. Although we plot species richness on the x-axis, we do not want to imply this direction for cause-effect. Please also note that there are no high-richness plots (> 17 species m^-2^) in Schorfheide-Chorin.

**Table S1**. Analysis of *total vegetation and soil carbon and nitrogen pools* in late 2009 (see Fig. 4 and 5). Data were log-transformed to achieve homogeneity of variances. Models were manually sim­plified until all model terms were significant. Design effects were always kept in the model.

|  |  | vegetation C | |  | vegetation N | |  | Soil C | |  | Soil N | |
| --- | --- | --- | --- | --- | --- | --- | --- | --- | --- | --- | --- | --- |
|  | df | SS | *F* |  | SS | *F* |  | *SS* | *F* |  | *SS* | *F* |
| Explo | 2 | 0.508 | 2.17^n.s.^ |  | 6.09 | 19.3*** |  | 39.9 | 227*** |  | 33.1 | 113*** |
| LUT | 2 | 0.0358 | 0.0179^n.s.^ |  | 0.277 | 0.877^n.s.^ |  | 0.087 | 0.494^n.s.^ |  | 0.293 | 0.996^n.s.^ |
| Treatment | 1 | 0.635 | 5.43* |  | 1.18 | 7.43** |  | 0.038 | 0.438^n.s.^ |  | 0.014 | 0.0937^n.s.^ |
| log(species richness) | 1 | 0.110 | 0.940^n.s.^ |  | 0.246 | 1.55^n.s.^ |  | 0.108 | 1.24^n.s.^ |  | 0.012 | 0.0783^n.s.^ |
| LUT:Explo | 4 | 2.88 | 6.15*** |  | 2.84 | 4.50** |  | 1.08 | 3.07* |  | 1.67 | 2.83* |
| Explo:log(species richness) | 2 |  |  |  |  |  |  |  |  |  | 1.71 | 5.81** |
| residuals |  | 8.65 | (df=74) |  | 11.7 | (df=74) |  | 6.57 | (df=75) |  | 10.7 | (df=73) |

Fig. S6. Outline of sub-plot design. The entire green area was covered by the OTC/rain shelter construction (or not, in controls). To reduce edge effects of this manipulation, all measurements were taken in the inner 1 x 2 m (dashed rectangle). Vegetation recording took place next to the biomass sampling area.

Fig. S7. Pictures of the OTC in winter (top), as rain shelter in summer (bottom right) and the visible effect of drought underneath (bottom left). (Photos by LvR.)
